# How are nature-based interventions defined in mild cognitive impairment and dementia studies? A conceptual systematic review and novel taxonomy

**DOI:** 10.1177/14713012241261788

**Published:** 2024-07-24

**Authors:** Harmony Jiang, Gill Eaglestone, Paul McCrone, Catherine Carr, Charlotte Stoner

**Affiliations:** Institute for Lifecourse Development, 411470 University of Greenwich, UK; Centre for Psychiatry and Mental Health, Wolfson Institute of Population Health, 339985 Queen Mary University of London, UK; Institute for Lifecourse Development, 411470 University of Greenwich, UK

**Keywords:** dementia, mild cognitive impairment, nature-based interventions, conceptual systematic review, taxonomy, nature

## Abstract

**Objectives:**

To systematically review research testing nature-based interventions for people living with mild cognitive impairment or dementia, and to report how authors have defined their interventions by presenting a taxonomy of the nature-based interventions.

**Methods:**

A conceptual systematic review of research published between 2008 and 2024 investigating nature-based interventions for people living with mild cognitive impairment or dementia was conducted. Three reviewers contributed independently. Exclusion criteria: not specifying if participants had mild cognitive impairment or dementia, only recruiting caregivers, no primary data, study protocols, abstracts, reviews, not peer-reviewed journal articles and any other grey literature. Intervention descriptions within the papers were thematically analysed.

**Results:**

Fifty-two articles reporting fifty-one studies were included. The most common interventions were nature virtual reality (VR technology) and gardening. From the definition data, we produced a taxonomy with overarching domains of: (a) Other terms used; (b) Characteristics; (c) Activities. Subdomains included: development or approach, modes of action, location, physical features, and activities. Some interventions could be grouped. Structure and standardisation of the interventions varied, with a lack of clear reporting.

**Conclusion:**

This taxonomy provides conceptualisations of nature-based interventions that can be used by future researchers to guide the development, evaluation and reporting of robust interventions in this area.

## Introduction

Nature-based interventions are structured programmes that employ nature-based experiences or initiatives, which aim to improve people’s health and wellbeing. Nature-based interventions can be both passive (e.g., viewing nature) and active (e.g., horticultural therapy) (Yeo et al., 2020). The positive effects of nature on health and wellbeing have been demonstrated in various populations, including in people living with mild cognitive impairment or dementia (Lu et al., 2020; Zagnoli et al., 2022)

Nature-based interventions for people living with mild cognitive impairment or dementia are an increasingly popular non-pharmacological therapeutic option (Natural England, 2016). Nature-based interventions can improve people living with mild cognitive impairment or dementia’s mood, wellbeing and quality of life, with effect on cognition being less conclusive (Edwards et al., 2013; Hewitt et al., 2013; Masuya & Ota, 2014; Pedrinolla et al., 2019; Scott et al., 2022). Post-diagnostic support for people living with mild cognitive impairment or dementia and caregivers is limited, with variation in services offered and specific groups being particularly disadvantaged, such as ethnic minority communities (Wheatley et al., 2021).

Nature-based interventions may be appealing for people living with mild cognitive impairment or dementia, as not only do humans have an instinctive affinity towards nature (Ulrich et al., 1991), this population may have more hobbies involving nature (e.g., gardening), due to changing life circumstances (Ball et al., 2007). However, nature-based interventions for people living with mild cognitive impairment or dementia constitute a broad field of research, encompassing a diverse group of interventions. Though more ‘traditional’ nature-based interventions, such as gardening, can be more readily classified, there are still differences in how researchers have conceptualised these. Furthermore, a lack of consensus on what each nature-based intervention constitutes makes it difficult to define what each nature-based intervention involves.

Varying definitions and unclear theoretical frameworks for defining nature-based interventions can lead to un-robust development of nature-based interventions, which is problematic as the interest into their therapeutic potential is growing (Nejade et al., 2022). Heterogeneity in describing nature-based interventions in clinical practice may also impact upon uptake by people living with mild cognitive impairment or dementia. Thus, we need to investigate how authors have defined nature-based interventions in mild cognitive impairment and dementia research, to identify any overarching conceptualisations of nature-based interventions and to create a taxonomy of nature-based interventions which can guide the future development of nature-based interventions and inform healthcare practitioners. To our knowledge, no other taxonomy has been presented for nature-based interventions for people living with mild cognitive impairment or dementia.

### Aims

1. To describe how specific nature-based interventions for people living with mild cognitive impairment or dementia have been defined in studies meeting our eligibility criteria, to highlight any overarching conceptualisations of nature-based interventions in previous literature.
2. To construct and present a novel taxonomy for different nature-based interventions for people living with mild cognitive impairment or dementia based on the eligible studies.

## Materials and methods

### Study design

We conducted a conceptual systematic review as it allows systematic exploration of variations of a term or idea (Ayala, 2018). According to Schreiber and Cramer (2022), a conceptual systematic review helps to ‘untangle a tangled term’, which in our case are the different nature-based interventions in dementia research, constituting several possible tangled terms. We incorporated review process elements from Schreiber and Cramer (2022) and Turin et al. (2020)’s paper on a systematic review protocol for taxonomy development. Reporting conformed to the Preferred Reporting Items for Systematic Reviews and Meta-Analyses (PRISMA) guidelines (Moher et al., 2015). There are no human participants in this article and neither informed consent nor ethical approval was required.

### Eligibility criteria

Primary research studies were included if they were published in English between 2008 and 2024, as this was a balanced timeframe for obtaining the most recent and relevant papers for investigating how authors define current nature-based interventions in dementia studies. Studies were also included if (i) Participants were 18 years old or over; (ii) At least 50% of participants had mild cognitive impairment or dementia (any subtype and stage, with or without a formal diagnosis); (iii) Any structured or standardised nature-based intervention was formally evaluated, by reporting (qualitative or quantitative) on the impact of the intervention on participants’ mental (including wellbeing, quality of life) and/or physical health. We included all study designs (apart from reviews), including randomised controlled trials, cross-sectional studies and pilot studies.

We excluded studies if they (i) Did not specify if the participants had dementia or mild cognitive impairment e.g. if authors stated participants were ‘older adults’ or ‘nursing home residents’; (ii) Only recruited caregivers; (iii) Did not report direct participant data; (iv) Were study protocols, conference abstracts, reviews (e.g. systematic, scoping, literature); (v) Were not peer-reviewed journal articles; (vi) Were any other grey literature.

### Data sources and search strategy

We identified eligible studies by searching the following databases: EBSCOhost (specifically MEDLINE, APA PsychInfo, CINAHL Plus with Full Text), PubMed and Cochrane Library. Google Scholar was searched and forward and backward citations of full-text articles included was conducted. Searches were last run on 20^th^ February 2024. No further papers were identified in this most recent search.

Search terms included phrases related to our population, intervention and outcome terms, namely ‘mild cognitive impairment’ or ‘dementia’, ‘nature’ and ‘effect of’ or ‘outcome’ (see Supplementary Table 1 for full list of search terms).

### Study selection

**Figure 1.**
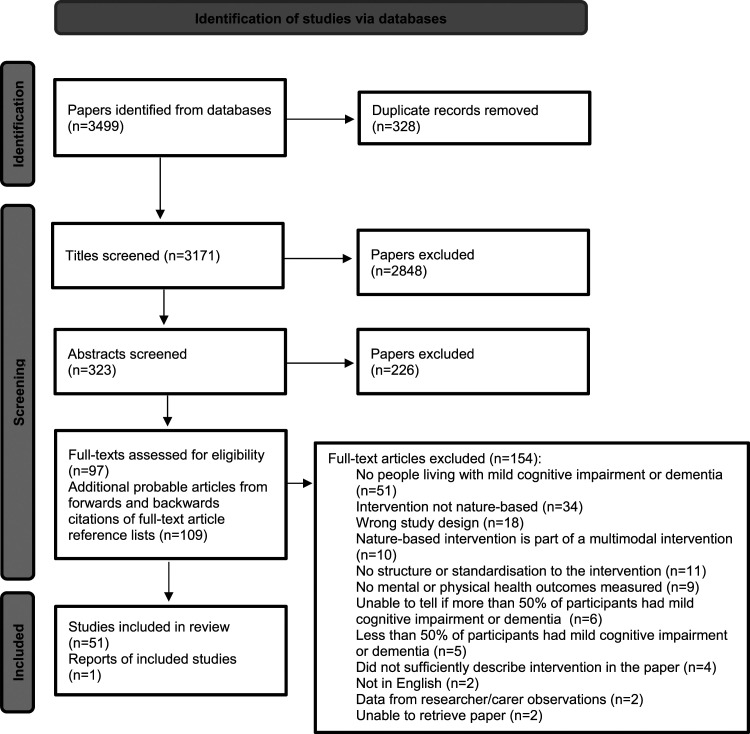
PRISMA flow diagram.

Firstly, papers from the database searches were downloaded into systematic review software Rayyan (Ouzzani et al., 2016). Titles of these papers were screened by the first reviewer using the eligibility criteria. Then, the abstracts of papers passing the title-screening stage were screened by the first and second reviewer. A third reviewer resolved any discrepancies. The full-texts of those passing the abstract-screening stage were screened by the first and second reviewer, with the third reviewer resolving any further discrepancies. The first reviewer contacted authors by email to request the full-texts for those we could not obtain. We obtained all the full-text papers apart from two. The first reviewer carried out forward and backward citations of the final group of full-text articles meeting our eligibility criteria to identify any other articles. All three reviewers worked independently. See Figure 1 for the PRISMA diagram.

### Data extraction

We extracted the following data from the final set of full-text papers, (i) Citation (Authors and year of publication); (ii) Nature-based intervention characteristics: type of nature-based intervention investigated, location of nature-based intervention, length of nature-based intervention, group, 1:1 or individual; (iii) Whether participants were recruited as a dyad or not; (iv) Primary and secondary outcome measures; (v) Main finding; (vi) Any descriptions about the intervention; (vii) Any descriptions about framework models or theories used to justify the author’s intervention. Any missing data has been described as “not stated in paper” in the descriptives table or is simply not listed in the taxonomy.

### Quality assessment

We did not assess the quality of the studies or risk of bias as we were not investigating the effectiveness of the interventions for our conceptual systematic review.

### Data synthesis

We followed a qualitative, thematic coding approach as proposed by Turin et al. (2020) to generate codes from the descriptions of nature-based interventions extracted from the papers. This enabled us to create a taxonomy for nature-based interventions in mild cognitive impairment and dementia research. The first reviewer extracted the descriptions about the nature-based interventions from the papers and inputted the data into NVivo [Release 1.7 1533 2022]. The first reviewer then read and re-read the extracted data to gain familiarity with the data, then carried out line-by-line, inductive, descriptive coding to create codes. Descriptive coding reports on what the data said, rather than interpreting the data (Linneberg & Korsgaard, 2019), which is relevant for our conceptual systematic review. The second reviewer ‘spot-coded’ excerpts from the extracted data to check if the coding was similar. Then the first reviewer carried out more focused coding to create categories, in which themes were developed. The codes, categories and themes were synthesised into a table which represented the taxonomy. See Supplementary Figure 1 for thematic analysis process.

## Results

A total of 3499 papers were identified from the initial database search, of which 328 were duplicates. Of these 3171 papers, 2848 were excluded at the title-screening stage and 226 were excluded at the abstract-screening stage, resulting in 97 papers for full-text screening. Forwards and backwards citations of the full-text article reference lists led to an additional 109 full-text papers screened. From these 206 full-text papers, 154 papers were excluded, resulting in a final 52 articles reporting 51 studies for analysis. See Figure 1 to see PRISMA diagram for reasons for exclusion. Table 1 shows a breakdown of nature-based interventions and number of articles investigating them.

**Table 1.** Nature-based interventions tested across the articles and the number of articles investigating them, in order of descending number of articles.

Nature-based intervention	Number of articles
Nature virtual reality (VR technology)	9
Gardening	9
Green care farm	8
Horticultural therapy	8
Garden visit	6
Japanese garden	3
Rice-farming care	3
Woodland activity programme	2
Beach simulation room	1
Forest bathing	1
Indoor gardening planting table game	1
Nature walk	1
Virtual nature experience (physical props)	1

### Characteristics of included articles

A comprehensive table describing each article is shown in Supplementary Table 3. Most of the studies were carried out in Western populations, with twenty-two studies being conducted in Europe, fifteen in North America, twelve in East Asia and three in Australia. The nature-based interventions were conducted in a mix of inpatient, care, residential and community settings depending on participants’ needs and resources required for the intervention. Most of the articles investigated group interventions (twenty-nine articles). One article tested two nature-based interventions, gardening and nature walk, with the same group of participants (Hendriks et al., 2016). Therefore, 52 articles were included in the review, however the total number of nature-based interventions included in Table 1 is 53.

### Grouping the nature-based interventions

**Figure 2.**
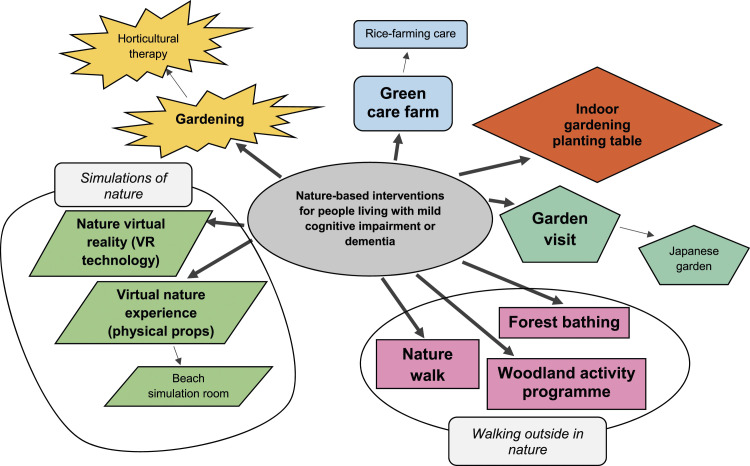
Diagram showing groupings of nature-based interventions in the studies reviewed, with smaller arrows indicating subtype.

Figure 2 demonstrates how the nature-based interventions could be grouped according to shared characteristics and activities. Horticultural therapy is a subtype of gardening as it is a more specialised form of gardening: horticultural therapy has a therapeutic goal and requires a qualified horticultural therapist. Similarly, rice-farming care is a subtype of green care farm as it is a more specialised form of it; it involves solely farming rice, as opposed to farming other plants. Japanese garden is a subtype of garden visit, as participants still visit the garden, but it is Japanese-themed. Woodland activity programme, nature walk and forest bathing are grouped as they all involve walking outside in nature.

Nature virtual reality (VR technology) and virtual nature experience (physical props) are grouped as their main mode of action is to immerse the participant in a simulation of nature. Beach simulation room is a subtype of virtual nature experience (physical props) as both use physical props to create the simulation, whereas nature virtual reality (VR technology) uses virtual reality technology. Indoor gardening planting table game is its own entity.

### Taxonomy of nature-based interventions from the reviewed articles

The taxonomy developed includes an overall definition of the nature-based intervention, derived from reviewing each of the articles grouped under a particular nature-based intervention. Then, from thematically analysing relevant data from the papers, three main domains were generated: other terms used (by authors to describe the nature-based intervention), characteristics (with subdomains of development or approach, modes of action, physical features, specific design features for people living with dementia, structured visits/sessions/programme) and activities (with categories of active and passive). See Table 2 for the full taxonomy.

**Table 2.** Taxonomy of nature-based interventions in the articles reviewed.

Nature-based intervention	Domain	Subdomain	Description
*Summary definition*			
Forest bathing	Other terms used	*Forest healing, forest therapy, natural therapy.*	
*An organised programme facilitated by a trained guide which incorporates a range of activities based in the forest.*	Characteristics	Development or approach	Developed by a ‘forest service’.
		Modes of action	Sharing and connecting with others, uses various physical and environmental factors of nature, holistic approach, immersive experience.
		Structured visits	A structured programme guided by trained instructors, with an overnight stay and meals provided.
	Activities	Active	Being mindful, meditation, cognitive training, learning about nutrition, walking, nature-themed activities.
Garden visit	Other terms used	*Enriched garden, improved garden, indoor therapeutic garden, sensory garden, wander garden, community garden.*	
*Structured and meaningful visits to gardens in a range of settings but most likely within a care facility, often making use of the multisensory experience in a garden setting.*	Characteristics	Development or approach	Collaboratively designed with a specialist, based on research, person-centred approach.
		Location	Inside a care facility, garden is already there or existing, can be an indoor or outdoor garden, separate from other areas, includes various settings such as in the community or inside a ward or hospital.
		Modes of action	Ability to walk around solo, free interaction with the garden, multisensory stimulation, sharing experiences with others, relaxation, staff encouragement to visit the garden, mimicking the outdoors.
		Physical features in the garden	Various features including flowers, plants, water features, places to sit, trees, shaded areas, shrubs, animals.
		Specific design features for people living with dementia	A number of adapted facilities aimed to make the garden safer and more accessible for people living with dementia such as specific access points to enter the garden, paths made with special material to prevent falls, continuous walking paths, non-toxic plants, having an alternative plan if the weather was bad.
		Structured visits	Visits to the garden are scheduled but range from 10-20 minute visits to 60 minute visits. Daily visits were most common. Staff accompanied the visits, with participants having the option to go with family or friends.
	Activities	Active	Engaging with the plants and flowers by touching, smelling and talking about them, doing some gardening, playing a game in the garden, reminiscing in the garden.
		Passive	Looking at the plants and flowers, sitting in the garden, eating or drinking in the garden.
Gardening	Other terms used	*Therapeutic gardening, indoor gardening, community gardening, adaptive gardening.*	
*A structured programme encompassing gardening activities, either inside or outside, both in a group and individually, with activities being flexible and adapted to the participant’s abilities and preferences.*	Characteristics	Development or approach	Activities were tailored to participant’s abilities, based on positive reinforcement, flexible and adaptive approach, research done to select which plants to include.
		Location	Outdoors or indoors depending on the weather.
		Modes of action	A mix of group and individual work, option to work by themselves or with others, participants could choose what to plant, ability to walk freely around the garden, staff engaging with and encouraging the participants to do gardening, multisensory experience, relaxation, opportunity to express and practice new skills learned.
		Physical features	Flowers, fruit trees, herbs, vegetable garden.
		Specific design features for people living with dementia	Modified gardening activities meeting the participant’s level of functioning, accompanied by trained staff.
		Structured sessions	A structured programme is followed of varying length and frequency of sessions. Sessions could be once or twice a week, for a month or 6 weeks. Participants helped to plan the sessions. Sessions are facilitated by specialists, trained staff, students and/or volunteers. Family members can join the sessions. Gardening equipment is typically provided.
	Activities	Active	Caring for and nurturing plants, cleaning, arranging containers and overall garden maintenance. Cooking with home-grown vegetables, participants providing feedback on the plants and written reflections.
Green care farm	Other terms used	*Farm-based day care*	
*An innovative type of 24 hour or day care where participants live and/or work together on a farm, helping with agricultural, work-related and domestic tasks.*	Characteristics	Development or approach	A type of ‘innovative’ adult day care predominantly developed in the Netherlands for a range of client groups, is empowerment-oriented and strengths-based.
		Location	A small-scale living facility either located on an existing, working farm in a non-institutional environment or as part of a nursing home.
		Modes of action	Opportunities to take part in various activities, activities are incorporated into daily life, in a group-setting facilitating social interaction, living together long-term, ability to move around the farm freely, a stimulating environment, using resources from the farm.
		Physical features	Imitation of a home-like environment, presence of animals, various outdoor areas such as farmyards, gardens, greenhouses and stables, a separate building as a base.
		Specific design features for people living with dementia	Offers respite for family caregivers, adapted facilities for the residents.
		Structured programme	A structured programme providing 24 hour or day care, facilitated by specially trained caregivers or staff who have integrated tasks, food provided.
	Activities	Active	A fusion of agricultural and care activities broadly split into ‘domestic activities’, ‘farm-related activities’ e.g. taking care of animals and gardening, and ‘work-related activities’ encompassing a mix of indoor and outdoor activities. Physical activity is prominent.
Horticultural therapy	Other terms used	*Horticultural-based activities, horticultural activities programme*	
*An ancient practice led by a horticultural specialist, nowadays following a manualised and evidence-based programme involving gardening-related activities with goals representing a holistic approach, carried out in a group-setting.*	Characteristics	Development or approach	Co-produced with specialists, strong-evidence base, an ancient practice, based on person-centeredness, holistic approach.
*A subtype of gardening (see* Figure 2 ).		Location	Indoors e.g. in a day care centre, or outdoors.
		Modes of action	Group-work, encouraging social interaction and a sense of community, participants can choose goals, engaging participants to learn new skills, opportunities for physical movement and to spend time outdoors, multisensory stimulation.
		Physical features	Raised garden beds, music played.
		Specific design features for people living with dementia	Safety preparation, alternative activities.
		Structured programme	Facilitated by specialists in horticultural therapy, manualised and evidence-based, structured, standardised programme, preparation involved such as preparing gardening equipment, activities are adapted to the season, sessions vary between 30-60 minutes, once or twice per week for 6–10 weeks.
	Activities	Active	Gardening-related activities, craft activities with a horticultural theme, garden party.
Indoor gardening planting table game	Characteristics	Modes of action	Multisensory and cognitive stimulation.
*A novel, indoor game incorporating cognitive and multisensory stimulation using gardening tasks.*	Activities	Active	Differing levels of gardening planting tasks.
Japanese garden	Other terms used	*Japanese-style garden, small or miniature garden, tea garden, interior garden, Japanese Zen garden, Japanese gardening.*	
*A specially constructed Japanese-style garden representing an abstraction of nature, and when constructed for people living with dementia or mild cognitive impairment is designed to be viewed indoors, encouraging the viewer to passively ‘feel the beauty’.*	Characteristics	Development or approach	A representation or abstraction of nature, an art.
*A subtype of garden visit (see* Figure 2 *).*		Location	Within a care facility, designed to be viewed from indoors, often temporarily installed.
		Modes of action	Is viewed only, a visual illusion using elements of nature, intimate relationship between the viewer and the garden, experiencing calmness and ‘feeling the beauty’.
		Physical features	Specially constructed, various natural objects such as bamboo screens, stone basin, stone lanterns, stepping stones, small maple tree, gravel path, nature sounds, no flowering plants, requires little maintenance.
		Specific design features for people living with dementia	Viewing the garden only, safety concerns considered.
		Structured visits	Viewed for 15 minutes, scheduled visits, with specific viewing points.
Nature virtual reality (VR technology)	Other terms used	*Virtual reality, virtual reality forest/garden, VR nature environment intervention, nature-based virtual reality environment, virtual reality platform, virtual reality therapy.*	
*An evidence-based approach co-produced by specialists, utilising virtual reality technology to provide an immersive nature experience, enabling the participant to ‘go outdoors’ and to interact with the virtual environment, creating a sense of presence and relaxation.*	Characteristics	Development or approach	Co-produced with specialists, interdisciplinary working with health professionals and researchers, evidence-based design, overcomes limitations of other therapies, unique experience or opportunity.
		Location	In an inpatient setting.
		Modes of action	Two primary types of virtual reality: computer-generated imagery (CGI) using video game technology and videos of real scenes of nature, both provide an immersive ‘artificial’ reality, enables ability to ‘go outdoors’ when not normally able to, multisensory experience, interactive, engaging, creates a sense of presence, relaxation.
		Physical features	Specialist equipment used, short video clips, nature scenes and sounds, 360-degrees, a ‘neutral’ environment, customisable material, motion, computer-generated, repeating or looping video.
		Specific design features for people living with dementia	Specially designed for people living with dementia or older adults, tested in a nursing home.
		Structured visits	One-off session, 13–30 minutes long, trained facilitator, with caregiver, participant made comfortable, good planning and preparation prior to the session.
	Activities	Active	Interacting with the video or virtual reality space.
		Passive	Viewing the video.
Nature walk	Characteristics	Mode of action	Connection between the facilitator and participants, multisensory stimulation, rest and relaxation, sense of calm.
*An organised walk in a natural outdoor setting, guided by a healthcare professional or volunteer, inducing a sense of calm, practicing multisensory stimulation, and carrying out cognitive tasks.*		Location	Outside.
		Specific design features for people living with dementia	Stimulation of memories, linking nature attributes to participant’s history.
		Structured sessions	Guided by a healthcare professional or volunteer.
	Activities	Active	Going through all the senses, asking questions.
Rice-farming care	Characteristics	Development or approach	Based on deeply culturally embedded behaviour and rituals, holds special cultural importance, community-based, based on the assumption that social participation improves wellbeing in elderly.
*A newly emerging community-based, ‘culturally specific’ nature-based intervention in East Asia which is based on rice-farming, involving a mix of physical and cognitive tasks surrounding rice planting and harvesting.*		Location	A rice field near a hospital.
*A subtype of green care farm (see* Figure 2 *)*		Modes of action	Working together in a group, reviewing the work, opportunity to express themselves, use of plants.
		Structured programme	A structured programme in a group, co-produced with people living with dementia, volunteers and staff, volunteers and staff providing assistance.
	Activities	Active	A mix of physical and cognitive tasks, rice planting and harvesting, caring and nurturing the plants, problem-solving tasks.
Simulated beach room	Other terms used	*Thematic multisensory stimulation.*	
*A 12-week, indoor beach-themed, multi-sensory stimulation group intervention facilitated by a nurse or occupational therapist.*	Characteristics	Modes of action	A positive theme, thematic interaction with others, multisensory stimulation.
*A subtype of virtual nature experience (physical props) (see* Figure 2 *).*		Physical features	A simulation of a beach environment, specialist equipment.
		Structured visits	30 minute sessions, 3 times a week for 12 weeks, in a group, visits were at the same time each day, facilitated by a nurse or occupational therapist.
Virtual nature experience (physical props)	Characteristics	Location	A memory care unit.
*A simulation of a nature experience based in a memory care unit, with a TV showing high-definition (HD) nature videos and nature-themed decorations, with the aim of promoting restoration from stress and a sense of immersion in nature.*		Modes of action	Restoration from stress, sense of presence or immersion.
		Physical features	Set up like a living room, TV showing HD videos, nature photos and sounds, plants.
		Structured visits	1 hour nature video.
Woodland activity programme	Characteristics	Development or approach	Co-developed, continually adapted or personalised.
*A personalised programme co-developed with rangers and participants, comprising a range of woodland-based activities where participants learn new skills and connect with others in a group-setting.*		Location	Local woodland.
		Modes of action	Connecting with others and sharing the experience, being present, stimulation of senses, supportive, empowering, relaxing, learning new skills.
		Specific design features for people living with dementia	Accessible woodland.
		Structured programme	A programme of activities facilitated by rangers, 4-week or 10-week programme, various activities.
	Activities	Active	Nature themed crafts/activities, walking, nature photography, writing and sharing poetry.
		Passive	Looking at nature, listening to birdsong.

### Nuances associated with terms used for nature-based interventions

Gardening and horticultural therapy as presented in our taxonomy were two separate, but linked, entities. They share substantial similarities, the main similarity being that both involve gardening activities. On the other hand, they also have differences: horticultural therapy has the overall goal of improving one’s health, whereas in gardening the goal is to grow and nurture plants. Horticultural therapy is facilitated by a qualified horticultural therapist who helps people progress towards therapeutic goals. Gardening is more informal and fundamentally focuses on the gardening work.

Two terms that were used interchangeably by authors of included articles were green care farms and farm-based day care. From the articles there were no obvious nuances in the characteristics and activities between the two, which was different to gardening and horticultural therapy as for these nature-based interventions authors often described their distinctions in their introduction or discussion. The absence of nuances in articles investigating ‘green care farms’ and ‘farm-based day care’ suggests that authors were naming the same nature-based intervention different terms. Therefore, we named any farm-based day care as a green care farm. Future researchers could do a more targeted comparison between green care farms and farm-based day care.

## Discussion

This paper aimed to synthesise and thematically analyse definitions of nature-based interventions for people living with mild cognitive impairment or dementia, to create an evidence-based taxonomy. Out of the 52 articles included, 13 nature-based interventions were identified, with nature virtual reality (VR technology) and gardening being the most popular.

### Nature-based interventions as a non-pharmacological dementia care intervention

As shown in Supplementary Table 3, most of the nature-based interventions tested in the articles resulted in positive outcomes for people living with mild cognitive impairment or dementia. Nature-based interventions have the potential to be a widely implementable and accessible non-pharmacological intervention for people living with mild cognitive impairment or dementia, to maintain or improve their mental and physical health. Our taxonomy can be used by researchers in the development of evidence-based nature-based interventions to ensure that interventions are developed using a sound theoretical framework. This is particularly important at a time where there is an increase in interest into the impact of nature and nature-based interventions on people’s health (Nejade et al., 2022).

### Taxonomies and their role in nature-based interventions and dementia care and research

Taxonomies can increase understanding of health interventions, including nature-based interventions, by offering a way to name and describe them which people can refer to (Bradley et al., 2007; Godfray & Knapp, 2004). Furthermore, taxonomies can help to present quite complex evidence-based information in a more accessible way. From our literature search, this is the first taxonomy presented for nature-based interventions for people living with mild cognitive impairment or dementia. Previous papers, such as Cousins et al. (2020) and Mohr et al. (2021), have aimed to categorise other interventions in dementia care, highlighting the variety of interventions within the dementia field. As the diversity of interventions for people living with mild cognitive impairment or dementia increases, researchers should seek to synthesise groups of these interventions into taxonomies, and clinicians and health professionals should utilise these taxonomies in their everyday conversations with people living with mild cognitive impairment or dementia. This will facilitate increased access to interventions as clinicians can draw on these taxonomies to better inform people living with mild cognitive impairment or dementia and caregivers, and information about interventions is more accessible for people living with mild cognitive impairment or dementia and caregivers.

### Methodological issues and limitations

Thematic analysis was well-suited for the purpose of this conceptual systematic review, though not every article had data for all the domains and subdomains. For example, in the taxonomy forest bathing is lacking information for several subdomains. This is likely because only one study was included for forest bathing, meaning there was not a lot of data for this nature-based intervention compared to others.

Another reason could be that the subdomains were not always relevant for each intervention. For example, gardening might not have any passive activities. This occurs in thematic analysis generally, such as whilst analysing interview data. For example, not every participant will give data for every interview question. Therefore we do not perceive this missing data as problematic.

In many articles, there was not enough information about the facilitator’s skills, specifically whether they had the necessary qualifications to be a horticultural therapist. Therefore, we had to categorise the nature-based interventions into either horticultural therapy or gardening based on what the authors described it as, even in the absence of detailed descriptions about the facilitator or intervention. Future studies should aim to state these details.

Another common issue in dementia research is not reporting the proportion of participants with dementia or mild cognitive impairment. Some of the papers, particularly those based in residential facilities, simply described the participants as having ‘cognitive decline’. This may represent the issue in nursing homes generally regarding the under-diagnosis of dementia and inconsistency in diagnoses across different nursing homes (Aldridge et al., 2023). Future researchers should aim to report this information.

Varying ‘interventions’ meant that it was sometimes difficult to decide which papers to include. For example, some studies tested the effect of nursing home residents using the garden, which we thought was too passive to be an intervention, compared to other initiatives such as a horticultural therapy programme. We decided to only include studies where there was some form of structure or standardisation to the intervention. For example, we would include a study if the intervention stated that it involved the staff encouraging the participants to visit the garden at least once a day, whereas we would not include a study if the participants could visit the garden whenever they wanted, as we argue that this is too passive, and should not be considered a nature-based intervention, but perhaps simply having access to nature.

Another methodological issue was not specifying the primary and secondary outcome measures, which should be standard practice in intervention research. Also, some papers did not mention theories or frameworks underpinning their intervention. Future researchers should include rationale to support their intervention, to ensure it is scientific.

### Implications for practice and future research

We propose that it is important for nature-based interventions to have two main components: structure and standardisation. Nature-based interventions should have some form of structure, which include number and duration of sessions, intervention length and session schedule/plan. Nature-based interventions should also be standardised, meaning that it should be delivered to everyone approximately the same, like in mindfulness-based interventions and exercise interventions. Therefore, we suggest that more passive forms of nature-based interventions, such as use of gardens without structure or standardisation, or access to green or blue space, do not constitute a nature-based intervention.

There was considerable heterogeneity in structure and standardisation within the same nature-based intervention across the articles reviewed. Future researchers should define a specific structure and standardisation for their nature-based intervention, to ensure that all participants are receiving the same intervention and to increase scientific integrity.

When assessing how the authors described their interventions, it was clear that even for the same nature-based intervention there were differences in the terms used, characteristics and activities. Due to these nuances, the taxonomy should be seen as an overall summary of the nature-based interventions included in this review. Future studies could develop taxonomies for each nature-based intervention and their subtypes, to give more detail and acknowledgement to the subtleties within the same nature-based intervention.

Some of the literature did not specify what their intervention entailed nor how it was developed. For example, some only described their intervention as a ‘non-pharmacological’ or ‘psychosocial’ intervention. We had to exclude these articles due to lack of information. Future authors should include detailed descriptions of evidence used to develop interventions and explicit details of the intervention being tested, to delineate if the intervention is nature-based, and to be able for future researchers to replicate the study.

Importantly, our taxonomy only represents studies that met our eligibility criteria, and the fifty-two articles do not represent the nature-based intervention field in mild cognitive impairment or dementia research altogether. However, the taxonomy can be used as a guide for researchers and healthcare professionals when researching or discussing nature-based interventions to their patients and caregivers. Strengths of the taxonomy include the definition summarising the nature-based intervention, which could be useful when discussing nature-based interventions in both a clinical and research setting. Another strength is the categories, as they are helpful to gain information about a specific attribute about a nature-based intervention. However, caution should be taken when using the taxonomy as it might not accurately represent nature-based interventions as a whole. Although we tried to keep our search strategy open to capture as many studies as possible, we still may have missed entire nature-based interventions where no studies met our eligibility criteria. Future studies could identify any nature-based interventions that we missed and add them to our taxonomy and publish a second version, to create a more comprehensive taxonomy.

## Conclusion

The field of nature-based interventions for people living with mild cognitive impairment or dementia is heterogeneous and complex. By systematically reviewing studies in this area, we developed a novel taxonomy containing conceptualisations of nature-based interventions in previous mild cognitive impairment and dementia studies. Our taxonomy can be used by future researchers to guide the development, evaluation and reporting of robust interventions for people living with mild cognitive impairment or dementia. Our taxonomy may also be useful in a clinical setting, to help healthcare professionals describe the different nature-based interventions to people living with mild cognitive impairment or dementia. Future research could seek to identify and add any nature-based interventions that may have not met our eligibility criteria at this time, and add detail to the nature-based interventions already present in our taxonomy.

## Supplemental Material

Supplemental Material - How are nature-based interventions defined in mild cognitive impairment and dementia studies? A conceptual systematic review and novel taxonomy Supplemental Material for How are nature-based interventions defined in mild cognitive impairment and dementia studies? A conceptual systematic review and novel taxonomy in Harmony Jiang, Gill Eaglestone, Paul McCrone, Catherine Carr and Charlotte Stoner in Dementia
