# Supplementary material for: How are nature-based interventions defined in mild cognitive impairment and dementia studies? A conceptual systematic review and novel taxonomy
Source: Dementia (London). 2024 Jul 24;24(3):480–505. doi: 10.1177/14713012241261788 (PMC11915771; doi:10.1177/14713012241261788)
Supplement: Supplemental Material - How are nature-based interventions defined in mild cognitive impairment and dementia studies? A conceptual systematic review and novel taxonomy [file sj-pdf-1-dem-10.1177_14713012241261788.pdf]

Supplementary material

Search strategy

The patient/population, intervention, comparison and outcomes (PICO) method (Richardson et al., 1995) was used in which:

- Population – people living with mild cognitive impairment or dementia (any subtype, any stage, with or without a formal diagnosis)
- Intervention – Nature-based interventions for people living with mild cognitive impairment or dementia either on their own or as a dyad with a caregiver
- Comparison – Not applicable for this review as it is a conceptual review and is not looking at effectiveness of interventions
- Outcome – Impact of the intervention on mental (including wellbeing and quality of life) and/or physical health

Supplementary Table 1. PICO search terms.

PICO	Key word	Alternatives
P	"Mild Cognitive Impairment", Dementia, Other possible words which could include MCI and PLWD	"mild cognitive impairment" OR MCI OR "cognitively impaired" OR "cognitive impairment" OR Dementia OR "Alzheimer's disease" OR "Alzheimer's dementia" OR "Alzheimers disease" OR "Alzheimers dementia" OR "frontotemporal dementia" OR "lewy body dementia" OR "dementia with lewy bodies" OR "vascular dementia" OR "demen*" OR senile OR amentia OR "Older adult" OR elderly
I	Nature (with all truncation and search terms)	Nature OR Nature-based OR "nature based" OR nature-assisted OR "nature assisted" OR "natural environment" OR "nature prescription" OR "nature-based recreation"

		OR “nature based recreation” OR “nature-based initiative” OR “nature based initiative” OR “nature therap*” OR outdoor OR “outdoor rehab*” OR “outdoor healthcare” OR “outdoor intervention” OR outside OR “open space” OR park OR mountain OR wilderness OR “wildlife” OR “wildlife program*” OR “wildlife therap*” OR countryside OR allotment OR wood* OR forest OR “forest bathing” OR vegetation OR tree OR green OR “green rehab” OR “green healthcare” OR “green intervention” OR “green exercise” OR “green prescription” OR greenness OR greenspace OR blue OR bluespace OR marine OR ocean OR sea OR lake OR river OR farm* OR horticultur* OR “therapeutic horticultur*” OR “social horticultur*” OR “sociohorticultur*” OR “horticultural therapy” OR garden* OR “therapeutic garden*” OR ecotherap* OR “adventure therap*” OR “Shinrin yoku”
O	Effect of/outcome	Effect* OR wellbeing OR well-being OR “well being” OR benefit* OR barrier* OR impact OR outcome* OR “quality of life” OR QoL OR mood OR stress OR depress* OR anxi* OR “mental health” OR psycholog* OR distress OR restorat* OR improve

Filters and limits used on databases

Supplementary Table 2. Filters and limits used on databases during the database searches.

**Database**	**Filters/limits used**
Cochrane Library (advanced search)	Title Abstract Keyword<br>In English<br>Trials<br>January 2008 – December 2023
EBSCOhost – Earth/Environment Databases, Health Sciences Databases, Life Sciences Databases, Psychology/Sociology Databases	Subject terms<br>In English<br>Peer-reviewed journal<br>Above 18<br>Humans<br>January 2008 – December 2023
PubMed (advanced search)	Title/Abstract<br>In English<br>Clinical Trial<br>RCT<br>Clinical Study<br>Observational Study<br>Clinical Trials<br>Comparative Study<br>Evaluation Study<br>January 2008 – December 2023
Google Scholar	2008 – 2023

Descriptives table of included articles

Supplementary Table 3. Descriptives of articles included in the systematic review.

Anderson et al. (2011)	
Type of nature-based intervention as defined by author	Garden visit (multisensory stimulation)
Location of intervention	Residential facility in Canberra, Australia
Length of intervention	3 sessions over 3 weeks
Group, 1:1 or individual?	1:1
Recruited as a dyad?	No – but family member/guardian provided proxy consent on participants' behalf
Primary outcome measure	Level of engagement and feasibility of implementing the Snoezelen programme into a long-term residential-aged care facility (primary and secondary outcome measures not specified)
Secondary outcome measure	None
Main finding	There was no significant difference between the Snoezelen room and garden for people living with dementia living in a residential facility. The interventions were difficult to implement in a residential facility setting.
Appel et al. (2021)	
Type of nature-based intervention as defined by author	Nature virtual reality
Location of intervention	Community teaching hospital in Toronto, Canada
Length of intervention	Option to spend up to 20 minutes at a time with virtual reality headset on (average 6.2 minutes) for 18 sessions
Group, 1:1 or individual?	Individual but carer often also sitting in with participants
Recruited as a dyad?	No - just people living with dementia recruited but carers helped with giving consent/assent
Primary outcome measure	Feasibility of the intervention, including tolerability, acceptability, comfort and safety
Secondary outcome measure	Frequency and intensity of behavioural and psychological symptoms of dementia, length of hospital stay, daily medication prescribed
Main finding	Virtual reality therapy was acceptable and feasible for people living with dementia admitted to an acute care

	ward.
Appel et al. (2024)	
Type of nature-based intervention as defined by author	Nature virtual reality
Location of intervention	Community teaching hospital in Toronto, Canada
Length of intervention	Up to 20 minutes, every 1-3 days
Group, 1:1 or individual?	Individual
Recruited as a dyad?	No - but some participant's substitute decision makers gave consent on their behalf
Primary outcome measure	Behavioural and psychological symptoms, falls, length of stay, quality of life
Secondary outcome measure	Acceptability, safety and patient experience of intervention
Main finding	Nature virtual reality therapy significantly reduced aggression and was acceptable and enjoyable for people living with dementia in acute care.
Baek et al. (2022)	
Type of nature-based intervention as defined by author	Forest bathing
Location of intervention	National Center for Forest Therapy, Yecheon-gun and Yeongju-si, South Korea
Length of intervention	One 2 day, 1 night session per week for 3 weeks
Group, 1:1 or individual?	Not clear from paper
Recruited as a dyad?	No
Primary outcome measure	Cognition, mood, quality of life, sleep, body composition, blood pressure, blood composition (primary and secondary outcomes not specified)
Secondary outcome measure	None

Main finding	The forest healing programme increased cognitive ability, decreased depression, improved sleep, reduced stress hormones and improved the functional fitness of people living with mild cognitive impairment.
Borella et al. (2023)	
Type of nature-based intervention as defined by author	Horticultural therapy (horticultural activities programme)
Location of intervention	A residential and daytime care facility in northern Italy
Length of intervention	12 x 40 minute sessions, which were delivered twice a week for 6 weeks
Group, 1:1 or individual?	Group
Recruited as a dyad?	Not clear from paper
Primary outcome measure	Cognitive functioning, mood, behavioural and psychological symptoms and quality of life (primary and secondary outcome measures not specified)
Secondary outcome measure	None
Main finding	People living with dementia who took part in the horticultural activities programme showed less behavioural and psychological symptoms and improved mood, with some also showing improvements in cognitive functioning and quality of life, compared to the control group.
Bourdon and Belmin (2021)	
Type of nature-based intervention as defined by author	Garden visit (enriched garden)
Location of intervention	Nursing homes (n=4) in France
Length of intervention	6 months during spring and summer. At least 4 visits to the garden per week
Group, 1:1 or individual?	Individual and 1:1
Recruited as a dyad?	No

Primary outcome measure	Cognition, activities of daily living, risk of falls (primary and secondary outcome measures not specified)
Secondary outcome measure	None
Main finding	Enriched gardens offer a new therapeutic approach for people living with dementia residing in retirement homes by providing stimulating psychomotor activities in a garden setting.
Collins et al. (2020)	
Type of nature-based intervention as defined by author	Garden visit (sensory garden, multisensory stimulation)
Location of intervention	Continuing care retirement community in a memory care centre, city/country not specified but authors from Clemson, South Carolina, USA
Length of intervention	30-45 minutes, 3 times a week for 4 weeks. In the mid-morning.
Group, 1:1 or individual?	Not clear from paper
Recruited as a dyad?	Yes – participant and family member
Primary outcome measure	Agitation and quality of life
Secondary outcome measure	None
Main finding	People living with dementia visiting the sensory garden had significantly reduced agitation and improvements in quality of life.
Cook (2020)	
Type of nature-based intervention as defined by author	Woodland activity programme
Location of intervention	Urban woodland in Scotland, UK
Length of intervention	10 weeks
Group, 1:1 or individual?	Group

Recruited as a dyad?	No
Primary outcome measure	Mental wellbeing
Secondary outcome measure	Community belonging
Main finding	People living with dementia who took part in the woodland activity programme reported increased feelings of self-worth, meaning and ability to maintain a sense of autonomy and identity.
D'Andrea et al. (2008)	
Type of nature-based intervention as defined by author	Horticultural therapy
Location of intervention	Dementia special care unit in a long-term care facility in New York, USA
Length of intervention	Twice a week for 12 weeks (24 sessions). Each session was 45 minutes.
Group, 1:1 or individual?	Group
Recruited as a dyad?	No – relatives or legal guardians signed consent form on behalf of participants
Primary outcome measure	Cognitive functioning
Secondary outcome measure	Participants' behaviour, social interaction and involvement in the therapy
Main finding	Horticultural therapy can help people living with moderate Alzheimer's disease in terms of helplessness, decision-making and socialisation.
de Boer et al. (2017a)	
Type of nature-based intervention as defined by author	Green care farm
Location of intervention	Green care farms (n=5) in south of the Netherlands
Length of intervention	No fixed length

Group, 1:1 or individual?	Group
Recruited as a dyad?	Not clear from paper
Primary outcome measure	Level of physical activity and social interaction
Secondary outcome measure	None
Main finding	The green care farm improved engagement and social interaction in people living with dementia, and they could be a viable alternative to traditional nursing homes.
de Boer et al. (2017b)	
Type of nature-based intervention as defined by author	Green care farm
Location of intervention	Green care farms (n=5) in south of the Netherlands
Length of intervention	No fixed length
Group, 1:1 or individual?	Group
Recruited as a dyad?	No – but legal representatives provided written informed consent. Residents provided assent to indicate willingness to take part in the study.
Primary outcome measure	Quality of care, quality of life and related outcomes (primary and secondary outcome measures not specified)
Secondary outcome measure	None
Main finding	Green care farms are a valuable alternative to nursing homes for people living with dementia.
de Bruin et al. (2010)	
Type of nature-based intervention as defined by	Green care farm

author	
Location of intervention	Green care farms (n=10) in the Netherlands
Length of intervention	6 hours per day, visits to the green care farm per participant varied per week
Group, 1:1 or individual?	Group
Recruited as a dyad?	No - primary family caregiver gave consent on behalf of people living with dementia
Primary outcome measure	Total dietary intake
Secondary outcome measure	None
Main finding	Green care farms promote improved dietary intake for people living with dementia residing in the community.
de Bruin et al. (2012)	
Type of nature-based intervention as defined by author	Green care farm
Location of intervention	Green care farms (n=15) in the Netherlands
Length of intervention	Varied
Group, 1:1 or individual?	Group
Recruited as a dyad?	No - primary family caregiver gave consent on behalf of people living with dementia
Primary outcome measure	Activities of daily living, total number of diseases, medication use (primary and secondary outcome measures not specified)
Secondary outcome measure	None
Main finding	Green care farms are not more effective than regular day care farms at maintaining functional performance in people living with dementia residing in the community.
de Bruin et al. (2015)	

Type of nature-based intervention as defined by author	Green care farm
Location of intervention	Green care farms (n=10) in the Netherlands
Length of intervention	Not reported
Group, 1:1 or individual?	Group
Recruited as a dyad?	Yes – people living with dementia and family caregiver
Primary outcome measure	Social participation
Secondary outcome measure	None
Main finding	Green care farms are valuable in terms of social participation for people living with dementia.
Ellingsen-Dalskau et al. (2021)	
Type of nature-based intervention as defined by author	Green care farm
Location of intervention	Green care farms (n=10) across various locations, Norway
Length of intervention	Not reported
Group, 1:1 or individual?	Group
Recruited as a dyad?	Not reported
Primary outcome measure	Quality of care
Secondary outcome measure	None
Main finding	Compared to regular day care, farm-based day care offers more activities for people living with dementia, which may improve various aspects of people living with dementia's lives.

Ferguson et al. (2020)	
Type of nature-based intervention as defined by author	Nature virtual reality
Location of intervention	Hospice, exact location not mentioned in paper but all authors are based in Nebraska, USA
Length of intervention	One-off session lasting up to 30 minutes
Group, 1:1 or individual?	Individual
Recruited as a dyad?	No but the participant's Power of Attorney provided assent
Primary outcome measure	Acceptability and tolerance of intervention, perceived experience of VR use, behavioural changes (primary and secondary outcome measures not specified)
Secondary outcome measure	None
Main finding	Virtual reality is a well-tolerated activity which can be meaningful and enjoyable for people living with dementia and their caregivers in a hospice setting
**Gibson et al. (2017)**	
Type of nature-based intervention as defined by author	Woodland activity programme
Location of intervention	Woodland in Bristol, UK
Length of intervention	4 sessions for 4 weeks, plus a tailored walk
Group, 1:1 or individual?	Group
Recruited as a dyad?	Yes
Primary outcome measure	Wellbeing and impact of the intervention
Secondary outcome measure	None
Main finding	People living with dementia who took part in the woodland activity programme reported feeling connected to nature, finding joy in learning and carrying out activities, and feeling inspired.

**Goto et al. (2014)**	
Type of nature-based intervention as defined by author	Japanese garden
Location of intervention	Nursing home in New Jersey, USA
Length of intervention	Twice a week for 15 minutes, for 4 weeks
Group, 1:1 or individual?	Individual (with research assistant present)
Recruited as a dyad?	No but consent was obtained through the family or a legally authorised representative
Primary outcome measure	Stress (primary and secondary outcome measures not specified)
Secondary outcome measure	None
Main finding	Exposure to a small interior Japanese garden may decrease stress and improve quality of life for people living with late-stage Alzheimer's disease.
**Goto et al. (2017)**	
Type of nature-based intervention as defined by author	Japanese garden
Location of intervention	General hospital in Japan (exact city not stated)
Length of intervention	Varied
Group, 1:1 or individual?	Varied
Recruited as a dyad?	No - but some participants (those living with more severe dementia) were consented by their legal guardian
Primary outcome measure	Eye movement, attention, behaviour and heart rate (primary and secondary outcome measures not specified)
Secondary outcome measure	None

Main finding	People living with middle-late stage dementia had significantly reduced heart rate and improved behavioural symptoms when viewing the Japanese garden when compared to the control group.
Goto et al. (2018)	
Type of nature-based intervention as defined by author	Japanese garden
Location of intervention	Hamano Hospital and Wakaba Terrace Nursing Home, Nagasaki, Japan
Length of intervention	15 minutes, twice per week, 5 weeks in total
Group, 1:1 or individual?	1:1 (with caregiver sitting with participant)
Recruited as a dyad?	No but if the participant did not have capacity to consent themselves, their legal guardian gave consent on their behalf
Primary outcome measure	Heart rate, behavioural symptoms (primary and secondary outcome measures not specified)
Secondary outcome measure	None
Main finding	Viewing a Japanese garden improved physiological stress and cognitive functions such as verbalisation and memory retrieval in people living with dementia.
Hall et al. (2018)	
Type of nature-based intervention as defined by author	Horticultural therapy
Location of intervention	McCormick Home (care home) in South-Western Ontario, Canada
Length of intervention	10 weeks
Group, 1:1 or individual?	Group
Recruited as a dyad?	No but if the participant was unable to provide their own consent, their power of attorney gave consent on

	their behalf
Primary outcome measure	Wellbeing, mood and engagement (primary and secondary measures not specified)
Secondary outcome measure	None
Main finding	People living with dementia who took part in horticultural therapy exhibited high levels of wellbeing, which remained after the programme finished.
Hendriks et al. (2016)	
Type of nature-based intervention as defined by author	Gardening and nature walk
Location of intervention	Various locations in the Netherlands
Length of intervention	Not reported
Group, 1:1 or individual?	Group
Recruited as a dyad?	Yes
Primary outcome measure	Acceptability, experience of and satisfaction with the intervention
Secondary outcome measure	None
Main finding	People living with dementia exhibited high levels of positive behaviour and low levels of negative behaviour during the interventions. All participants enjoyed the activities and said they would do them again.
Hewitt et al. (2013)	
Type of nature-based intervention as defined by author	Gardening
Location of intervention	Thrive Trunkwell Garden Project in Berkshire, UK and Barkham Day Hospital garden in Wokingham Community Hospital, Berkshire, UK (community)

Length of intervention	2 hours per week, for 46 sessions
Group, 1:1 or individual?	Group
Recruited as a dyad?	Yes - participant and carer
Primary outcome measure	Wellbeing
Secondary outcome measure	Cognitive functioning of people living with young-onset dementia and carer perceptions of the intervention
Main finding	A gardening programme can maintain or improve wellbeing in people living with young-onset dementia.
**Hsieh et al. (2022)**	
Type of nature-based intervention as defined by author	Nature virtual reality
Location of intervention	Nursing homes (n=2) in New Taipei City, Taipei, Taiwan
Length of intervention	One-off 6 minutes (viewing the virtual reality video)
Group, 1:1 or individual?	Individual
Recruited as a dyad?	No
Primary outcome measure	Heart rate and heart rate variability
Secondary outcome measure	None
Main finding	Nursing homes can provide immersive experiences which can help to improve heart rate variability, memory recall and quality of life of their residents living with mild-moderate cognitive impairment.
**Ibsen et al. (2020)**	
Type of nature-based intervention as defined by author	Green care farm

Location of intervention	Green care farms (n=30) in various locations in Norway
Length of intervention	Varied
Group, 1:1 or individual?	Group
Recruited as a dyad?	Yes – people living with dementia and next of kin
Primary outcome measure	Characteristics of participants, farm characteristics and quality of life (primary and secondary outcomes not specified)
Secondary outcome measure	None
Main finding	Time spent outdoors was an important factor in reported quality of life in farm-based day care for people living with dementia. Participants attending farm-based day care in this study tended to be people living with early-stage dementia, and were more likely to be men with good physical and mental health.
Ibsen and Eriksen (2021)	
Type of nature-based intervention as defined by author	Green care farm
Location of intervention	Green care farms (n=5) in various locations in Norway
Length of intervention	Varied
Group, 1:1 or individual?	Not clear from paper
Recruited as a dyad?	No
Primary outcome measure	How people living with dementia describe attending farm-based day care
Secondary outcome measure	None
Main finding	Farm-based day care provides person-centred care and could be provided as a more active addition to regular day care for people living with dementia.
Jarrott et al. (2010)	
Type of nature-based	Horticultural therapy

intervention as defined by author	
Location of intervention	4 treatment sites at nursing home and advanced directives (an advance care planning tool) in rural southwest Virginia, USA
Length of intervention	Twice a week for 6 weeks
Group, 1:1 or individual?	Group
Recruited as a dyad?	No
Primary outcome measure	Engagement and affect (primary and secondary outcome measures not specified)
Secondary outcome measure	None
Main finding	Horticultural therapy improved engagement and helped to decrease distress and behavioural symptoms in people living with dementia.
Kalantari et al. (2022)	
Type of nature-based intervention as defined by author	Nature virtual reality
Location of intervention	Study site in Ithaca, New York, USA
Length of intervention	30 minute session
Group, 1:1 or individual?	Individual
Recruited as a dyad?	No
Primary outcome measure	Mood, attitudes towards virtual reality technologies (primary and secondary outcome measures not specified)
Secondary outcome measure	None
Main finding	No differences were found in the outcome measures in participants living with or without cognitive impairment.

Kim et al. (2020)	
Type of nature-based intervention as defined by author	Horticultural therapy
Location of intervention	Health centre in H-gun, Gyeongsangnam-do, South Korea
Length of intervention	15 x 100 minute sessions (Longer Treatment group = once a week for 15 weeks and Shorter Treatment Group = twice a week for 7.5 weeks)
Group, 1:1 or individual?	Group
Recruited as a dyad?	Yes - participant and their guardian
Primary outcome measure	Cognitive function, depression and life satisfaction (primary and secondary outcome measures not specified)
Secondary outcome measure	None
Main finding	Life satisfaction significantly improved, and depression significantly decreased in people living with mild cognitive impairment or mild dementia who took part in the horticultural therapy groups. No significant change in cognitive function in either group.
**Lassell et al. (2021)**	
Type of nature-based intervention as defined by author	Gardening
Location of intervention	Local senior centre in Northern Colorado, USA
Length of intervention	1 hour per week for 8 weeks
Group, 1:1 or individual?	Group
Recruited as a dyad?	No - but family member gave consent on behalf if needed to
Primary outcome measure	Quality of life
Secondary outcome measure	None
Main finding	Adaptive gardening and adaptive riding supported quality of life, whilst adaptive riding supported active participation for longer and those living with more complex dementia.

**Lee and Kim (2008)**	
Type of nature-based intervention as defined by author	Gardening
Location of intervention	Institution, exact location not stated but authors are from Jeonbuk, South Korea
Length of intervention	Twice a day, for 1 hour, for 4 weeks
Group, 1:1 or individual?	1:1
Recruited as a dyad?	No - but family member gave consent on behalf if needed to
Primary outcome measure	Sleep, agitation and cognition (primary and secondary outcome measures not specified)
Secondary outcome measure	None
Main finding	Indoor gardening improved sleep, agitation and cognition of people living with dementia.
**Masuya and Ota (2014)**	
Type of nature-based intervention as defined by author	Horticultural therapy
Location of intervention	Long-term care facility, location not stated but authors are from Japan
Length of intervention	30-40 minute sessions, once a week for 6 consecutive weeks
Group, 1:1 or individual?	Group
Recruited as a dyad?	No
Primary outcome measure	Vitality and cognitive function (primary and secondary outcome measures not specified)
Secondary outcome measure	None
Main finding	Horticultural therapy significantly improved vitality and cognition in people living with dementia.

**Mitchell and Van Puymbroeck (2019)**	
Type of nature-based intervention as defined by author	Gardening
Location of intervention	Long-term care setting, location not stated but author is from Clemson, South Carolina, USA
Length of intervention	40-60 minute sessions, 3-4 times per week for 6 weeks (17 sessions in total)
Group, 1:1 or individual?	1:1
Recruited as a dyad?	Not reported
Primary outcome measure	Anxiety and depression
Secondary outcome measure	None
Main finding	Gardening significantly decreased depression and anxiety in people living with dementia.
**Morris et al. (2021)**	
Type of nature-based intervention as defined by author	Gardening
Location of intervention	Dementia hub (community), location not specified but authors from Salford, UK
Length of intervention	8 sessions (a further planned 8 were cancelled due to COVID-19 lockdown)
Group, 1:1 or individual?	Group
Recruited as a dyad?	Yes - with spouse or family member. 6 were former care partners so were not recruited as a dyad
Primary outcome measure	Wellbeing
Secondary outcome	None

measure	
Main finding	Gardening improved mood and wellbeing in people living with dementia.
Motealleh et al. (2022)	
Type of nature-based intervention as defined by author	Garden visit (improved garden)
Location of intervention	Residential aged care facility in Brisbane, Australia
Length of intervention	60 minute sessions, 5 days per week for 4 weeks
Group, 1:1 or individual?	1:1
Recruited as a dyad?	No
Primary outcome measure	Agitation, engagement and apathy (all stated as primary outcome variables)
Secondary outcome measure	Activities in the garden carried out, participant perceptions of the garden
Main finding	The improved garden improved engagement and decreased apathy and agitation in people living with dementia living in a residential aged care facility.
Moyle et al. (2018)	
Type of nature-based intervention as defined by author	Nature virtual reality
Location of intervention	Residential aged care facilities (n=2) in Victoria, Australia
Length of intervention	One-off 15 minute session
Group, 1:1 or individual?	Individual
Recruited as a dyad?	No - but for most of the people living with dementia, consent was obtained via their legal guardians on their behalf

Primary outcome measure	Engagement, apathy and mood
Secondary outcome measure	Experiences of the intervention of people living with dementia, families and staff
Main finding	Nature virtual reality may improve quality of life in people living with dementia but there was greater level of fear/anxiety during the nature virtual reality experience.
**Noone and Jenkins (2018)**	
Type of nature-based intervention as defined by author	Gardening
Location of intervention	Community hall garden in Glasgow, Scotland, UK
Length of intervention	Weekly
Group, 1:1 or individual?	Group
Recruited as a dyad?	No
Primary outcome measure	Participants' experience
Secondary outcome measure	None
Main finding	Gardening can promote social citizenship and self-expression, as well as promoting social interaction and outdoor engagement in people living with dementia.
**Orr et al. (2021)**	
Type of nature-based intervention as defined by author	Nature virtual reality
Location of intervention	Memory cafés (n=2) in Cornwall, England, UK
Length of intervention	One-off session showing 5 x 30 second video clips

Group, 1:1 or individual?	Individual
Recruited as a dyad?	Yes – with their carer
Primary outcome measure	Experience of intervention for participants and carer and staff perception of intervention
Secondary outcome measure	None
Main finding	The virtual reality nature experience offered the opportunity for people living with cognitive impairment to be immersed in nature and enhanced their quality of life.
Pedrinolla et al. (2019)	
Type of nature-based intervention as defined by author	Garden visit (indoor therapeutic garden)
Location of intervention	Therapeutic garden in a retirement home in Mantua, Italy
Length of intervention	2 hours, 5 times a week for 6 months (120 sessions, 240 hours)
Group, 1:1 or individual?	Group
Recruited as a dyad?	No
Primary outcome measure	Behavioural and psychological symptoms
Secondary outcome measure	Use of medications for behavioural and psychological symptoms, cognition, activities of daily living, salivary cortisol and blood pressure
Main finding	Indoor therapeutic garden reduced behavioural and psychological symptoms, medication intake and cortisol levels in people living with Alzheimer’s disease.
Reynolds et al. (2018)	
Type of nature-based intervention as defined by author	Virtual nature experience

Location of intervention	Memory care unit within an assisted living facility, location not stated but authors from Arizona, USA
Length of intervention	1 hour video, 3 times
Group, 1:1 or individual?	Individual
Recruited as a dyad?	No - but consent given by legally authorised representative of participant on their behalf
Primary outcome measure	Heart rate, emotions and agitation (primary and secondary outcome measures not specified)
Secondary outcome measure	None
Main finding	Virtual nature experience reduced stress and negative emotions, and increased pleasure in people living with dementia.
Rose et al. (2021)	
Type of nature-based intervention as defined by author	Nature virtual reality
Location of intervention	Specialist inpatient psychiatric care setting in the UK (exact city not specified)
Length of intervention	Up to 15 minutes
Group, 1:1 or individual?	Individual
Recruited as a dyad?	No - but some participants were consented by a consultee if they did not have capacity to consent themselves
Primary outcome measure	Affect, wellbeing and behaviour (primary and secondary outcome measures not specified)
Secondary outcome measure	None
Main finding	Nature virtual reality is feasible for those living with mild-moderate stage dementia in an inpatient psychiatric care setting.
Shen et al. (2022)	
Type of nature-based	Horticultural therapy

intervention as defined by author	
Location of intervention	Day care centre in Taichung, Taiwan
Length of intervention	6 x 1 hour sessions
Group, 1:1 or individual?	Group
Recruited as a dyad?	No
Primary outcome measure	Psychological state, sleep quality and salivary markers of mucosal immunity
Secondary outcome measure	None
Main finding	Horticultural therapy significantly improved mood, satisfaction, wellbeing, sleep quality and increased mucosal immune proteins in people living with mild cognitive impairment or dementia.
Smith-Carrier et al. (2019)	
Type of nature-based intervention as defined by author	Gardening
Location of intervention	Adult day centre in Southwestern Ontario, Canada
Length of intervention	4 months (6 x 3 day 'waves' of activities with a break in between the waves)
Group, 1:1 or individual?	Group
Recruited as a dyad?	Yes
Primary outcome measure	What gardening meant to the participants in terms of the gardening process and experience<br>(primary and secondary outcome measures not specified)
Secondary outcome measure	None
Main finding	Important themes surrounding outdoor gardening included activation of the senses, being occupied in meaningful work, creating a sense of wonder and curiosity, learning new skills, socialisation, being present and mental and physical wellbeing in people living with dementia.

Styck and George (2022)	
Type of nature-based intervention as defined by author	Gardening
Location of intervention	Grand Pines Assisted Living Center (a residential living community) in Michigan, USA
Length of intervention	1 hour, twice a week for 2 months (18 sessions in total)
Group, 1:1 or individual?	Group
Recruited as a dyad?	Some yes, others no
Primary outcome measure	Sense of purpose
Secondary outcome measure	Quality of life
Main finding	Gardening was shown to have biopsychosocial benefits and improved sense of purpose for people living with dementia.
**Tseng et al. (2020)**	
Type of nature-based intervention as defined by author	Indoor gardening planting table game
Location of intervention	A day care centre in Douliu City, Taiwan
Length of intervention	5 weeks
Group, 1:1 or individual?	Group and 1:1
Recruited as a dyad?	Yes – with their families
Primary outcome measure	Cognitive performance
Secondary outcome measure	None
Main finding	After the indoor gardening planting table game intervention, cognition improved with low effect size, in people living with mild-moderate dementia.

Ura et al. (2018)	
Type of nature-based intervention as defined by author	Rice-farming care
Location of intervention	Rice farm, location not specified but authors from Japan
Length of intervention	Once every week for 25 weeks
Group, 1:1 or individual?	Group
Recruited as a dyad?	Not reported
Primary outcome measure	Wellbeing
Secondary outcome measure	None
Main finding	Rice-farming care improved social participation and wellbeing in people living with cognitive impairment.
Ura et al. (2021)	
Type of nature-based intervention as defined by author	Rice-farming care
Location of intervention	A rice field 10 minutes' walk from the hospital, location not specified but authors from Tokyo, Japan
Length of intervention	One hour per week, for 25 weeks
Group, 1:1 or individual?	Group
Recruited as a dyad?	No
Primary outcome measure	Experience of intervention, cognitive ability, mental wellbeing (primary and secondary outcome measures not specified)
Secondary outcome measure	None

Main finding	Rice-farming care significantly improved wellbeing but did not significantly improve cognitive function in people living with dementia when compared to the control group.
van der Velde-van Buuringen et al. (2021)	
Type of nature-based intervention as defined by author	Garden visit (daily garden use)
Location of intervention	Nursing home in the Netherlands
Length of intervention	At least 30 minutes every day for 2 weeks
Group, 1:1 or individual?	1:1
Recruited as a dyad?	No – but a legal representative gave consent on behalf of participants
Primary outcome measure	Usefulness, feasibility and applicability of the intervention and quality of life and neuropsychiatric symptoms
Secondary outcome measure	Number of falls, use of psychotropic medication and type of outdoor activities
Main finding	Daily garden visits increases positive affect and decreases social isolation in people living with dementia.
Verkaik et al. (2019)	
Type of nature-based intervention as defined by author	Beach simulation room
Location of intervention	A nursing home in Amsterdam in the Netherlands
Length of intervention	3 times a week for 30 minutes, for 12 weeks
Group, 1:1 or individual?	Group
Recruited as a dyad?	No - but participants were recruited by their legal guardian on their behalf
Primary outcome measure	Depression, agitation and apathy
Secondary outcome	Sleep-wake patterns, observed behaviour and mood

measure	
Main finding	The beach simulation room did not significantly reduce psychological and behavioural symptoms in people living with dementia in a nursing home when compared to a control group.
Walden and Faliciano (2021)	
Type of nature-based intervention as defined by author	Nature virtual reality
Location of intervention	Long-term facility (memory care unit) in Southern Colorado, USA
Length of intervention	One-off session that ranged from 3 to 11 minutes
Group, 1:1 or individual?	Individual
Recruited as a dyad?	No - participant's medical power of attorney consented them on their behalf
Primary outcome measure	Agitation
Secondary outcome measure	None
Main finding	Nature virtual reality decreased agitation in people living with dementia.
Yamazaki et al. (2019)	
Type of nature-based intervention as defined by author	Rice-farming care
Location of intervention	A rice field, location not stated but authors from Japan
Length of intervention	90 minute session, once a week for 25 weeks
Group, 1:1 or individual?	Group
Recruited as a dyad?	Not reported

Primary outcome measure	Cognitive function and mental health
Secondary outcome measure	None
Main finding	Rice-farming care led to improved cognitive function and mental health at the 6-month follow-up in people living with cognitive impairment.
Yang et al. (2022)	
Type of nature-based intervention as defined by author	Horticultural therapy
Location of intervention	Nursing home in Guangzhou, China
Length of intervention	60 minute session, once a week for 10 weeks
Group, 1:1 or individual?	Group
Recruited as a dyad?	No - but family member gave consent on behalf
Primary outcome measure	Apathy
Secondary outcome measure	Cognitive function, quality of life and functional capacity
Main finding	Horticultural therapy reduced apathy and increased cognitive function, but there were no effects on quality of life and functional capacity in people living with dementia.

Thematic analysis process.

Adapted from Turin et al. (2020)'s systematic review protocol towards developing a taxonomy of definitions, frameworks and methods in community engagement with immigrant communities involving health and wellness research.

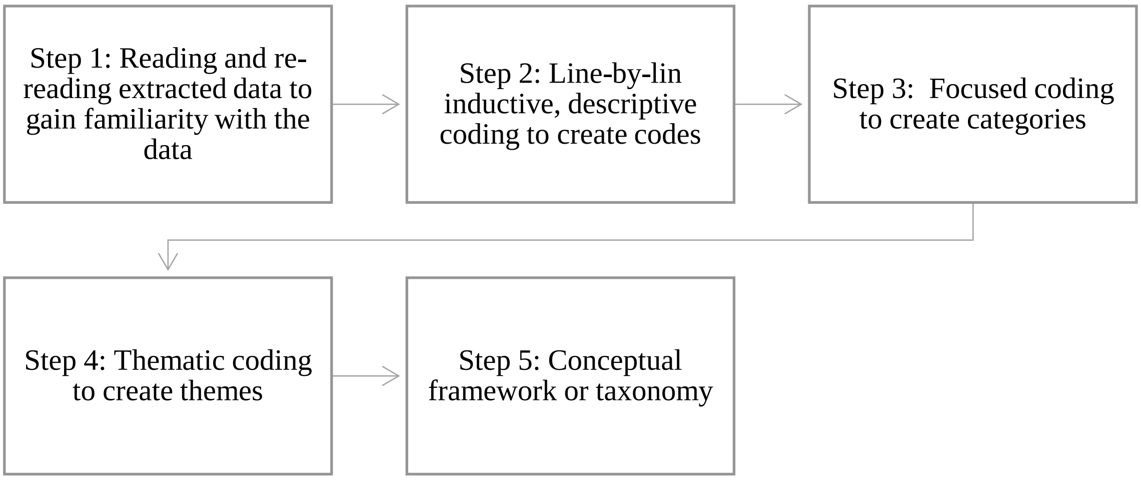

Supplementary Figure 1. Thematic analysis process, adapted from Turin et al. (2020).

Overview of domains and subdomains in the taxonomy, including descriptions

Supplementary Table 4. Domains and subdomains of the taxonomy, with descriptions of subdomains.

Domain	Subdomain	Descriptions
Other terms used	N/A	Any other ways the authors described the nature-based intervention in the paper.
Characteristics	Development or approach	Ways in which the nature-based intervention was designed, any psychological/philosophical/physiological/social approaches which the nature-based intervention is based on.
	Location	The setting and location where the nature-based intervention was delivered.
	Modes of action	Mechanisms of change of the nature-based intervention in regards to the participant's mental and/or physical health.
	Physical features	Objects that were involved in the nature-based intervention, whether used as a design feature or naturally occurring.
	Specific design features for people living with dementia	Alterations or adjustments in the nature-based intervention, most commonly in the physical space or structure of the sessions, to make the nature-based intervention more accessible and safer for people living with dementia.
	Structured session/visit/programme	The length, timing and frequency of the nature-based intervention, also details about the facilitators, any equipment/food provided and how the sessions were planned.
Activities	Active	Any activities in the nature-based intervention in which the participant consciously engages with and provides

		input into.
	Passive	Any activities in the nature-based intervention in which the participant does not consciously engage with, is more sedentary.

Abbreviations

Supplementary Table 5. Abbreviations within the paper and their expansions.

CGI	Computer-generated imagery
HD	High-definition
N/A	Not applicable
PICO	Patient/population, intervention, comparison and outcomes
PRISMA	Preferred Reporting Items for Systematic Reviews and Meta-Analyses
VR	Virtual reality
